# A Rare Complication of Thoracic Spine Surgery: Pediatric Horner’s Syndrome after Posterior Vertebral Column Resection—A Case Report

**DOI:** 10.3390/children10010156

**Published:** 2023-01-13

**Authors:** Pawel Grabala, Kinga Danowska-Idziok, Ilkka J. Helenius

**Affiliations:** 1 University Children’s Hospital, Department of Pediatric Orthopedic Surgery and Traumatology, Medical University of Bialystok, Waszyngtona 17, 15-274 Bialystok, Poland; 2 Paley European Institute, Al. Rzeczypospolitej 1, 02-972 Warsaw, Poland; 3 Department of Orthopedics and Traumatology, Helsinki University Hospital, 00260 Helsinki, Finland

**Keywords:** pediatric Horner’s syndrome, congenital scoliosis, complications of spine surgery, vertebral column resection, vcr

## Abstract

Background: Horner’s syndrome (HS) classically consists of the symptom triad of miosis, ptosis, and anhidrosis. It is caused by impairment of a certain pathway in the sympathetic nervous system. It may also appear as part of the clinical signs of other diseases and syndromes, including Pancoast tumors, intradural and/or epidural tumors, thoracic outlet syndrome, syringomyelia, brachial plexus injury, and aortic dissection. Here, we report a very rare complication of vertebral column resection in children, and we present the clinical findings of a case of Horner’s syndrome with a current literature review. Case presentation: A five-year-old child with severe congenital kyphoscoliosis qualified for surgical treatment of the spinal deformity via a posterior approach, with three-column osteotomy and fusion. Results: After successful surgery, the patient presented with HS due to distraction of the sympathetic nerve trunk and, thus, innervation to the left eye. At the 4-year follow-up, the child had fully recovered. Conclusions: Pediatric HS after posterior instrumented scoliosis correction surgery with posterior vertebral column resection of the thoracic spine is very rare. This is the first reported case of HS after posterior vertebral column resection and spinal fusion for congenital kyphoscoliosis without the use of epidural analgesia. Symptom resolution may be variable and, in some cases, delayed.

## 1. Introduction

Horner’s syndrome (HS) classically consists of the symptom triad of miosis, ptosis, and anhidrosis [1]. It is associated with damage to a specific pathway in the sympathetic nervous system, and it manifests as clinical symptoms resulting from the interruption of the neural pathway from the brain to the face and eye on one side of the body. The visible symptoms result from an interruption of sympathetic innervation of the eye, which can manifest on many different levels [2]. The damage may be located anywhere in the 3-neuron pathway [3,4,5]. Symptoms may also manifest in other diseases and clinical syndromes, such as thoracic outlet syndrome, brachial plexus injury, Pancoast tumors, intradural and/or epidural tumors, aortic dissection, and syringomyelia [5,6,7,8]. Few researchers have reported that HS may occur as a complication of the surgical treatment of other spinal pathologies or cervical spine surgery [9,10,11]. In the available scientific literature, there are reports of HS as a symptom occurring in the course of disc herniation of the thoracic spine and high intervertebral disc herniation. This symptom may appear after surgeries of the anterior cervical spine due to intraoperative damage to the cervical sympathetic trunk [9,10,11]. Even then, it is not treated by spine surgeons as a complication because blepharoptosis can be disgorged and cause visual impairment. The patient may also complain of a stuffy nose and ipsilateral nasal congestion [8,12,13,14].

Based on scientific reports, the incidence of HS as a complication of cervical spine surgery is quite high after anterior cervical discectomy, ranging from 0.06% to 3.8%. Some researchers have noted a higher rate (as high as 57%) of HS with oblique corpectomy after an anterolateral approach to the cervical spine and after revision surgery [3,4,5]. In contrast, full recovery is estimated at around 80–100% within six months of the onset of HS complications [4,5,6,7,8,9,10,11,12,13,14].

The purpose of this work was to present a case report of a very rare complication after posterior spinal surgery in a pediatric patient with a severe congenital spinal deformity.

## 2. Case Report

A five-year-old child was referred to our spine unit for surgical treatment of a severe congenital spinal deformity of malformed vertebrae and failure of segmentation at the cervical and thoracic levels. A congenital spinal defect was diagnosed before the age of one by a pediatrician who referred the child for outpatient treatment to a pediatric orthopedist. Initially, the child was treated conservatively, with observation and rehabilitation. The curvature of the spine progressed as the child grew. At around age four, the curvature progressed most rapidly. Attempts to treat with a corset were ineffective, and the only option for further treatment was surgery.

**Figure 1:**
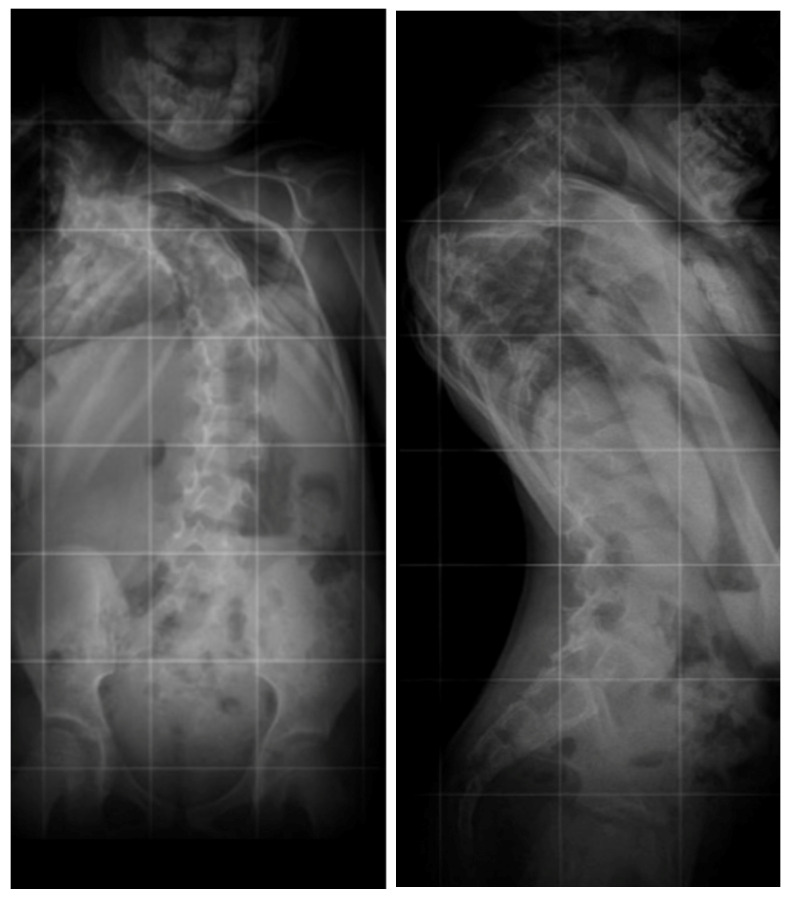
Standing X-rays of the five-year-old girl before surgical treatment.

After a thorough orthopedic examination and additional tests (X-ray, MRI of the entire spine, CT), and consultations with other specialists, including a cardiologist, pulmonologist, neurologist, and neurosurgeon, the five-year-old female pediatric patient with congenital scoliosis and severe kyphoscoliosis qualified for surgical treatment of the spinal deformity via a posterior approach with three-column osteotomy and fusion (Figure 1).

**Figure 2:**
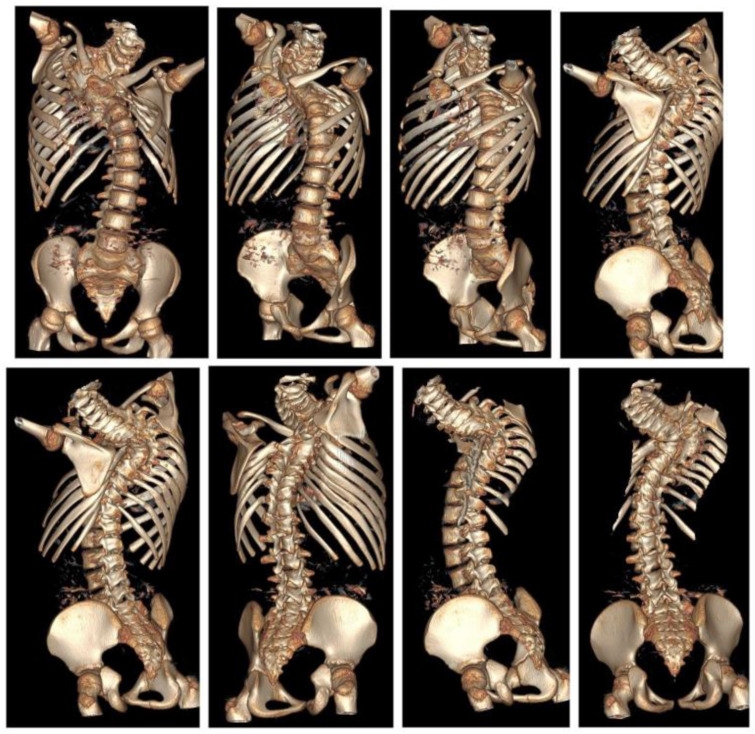
3D computed tomography of the five-year-old girl before surgical treatment.

**Figure 3:**
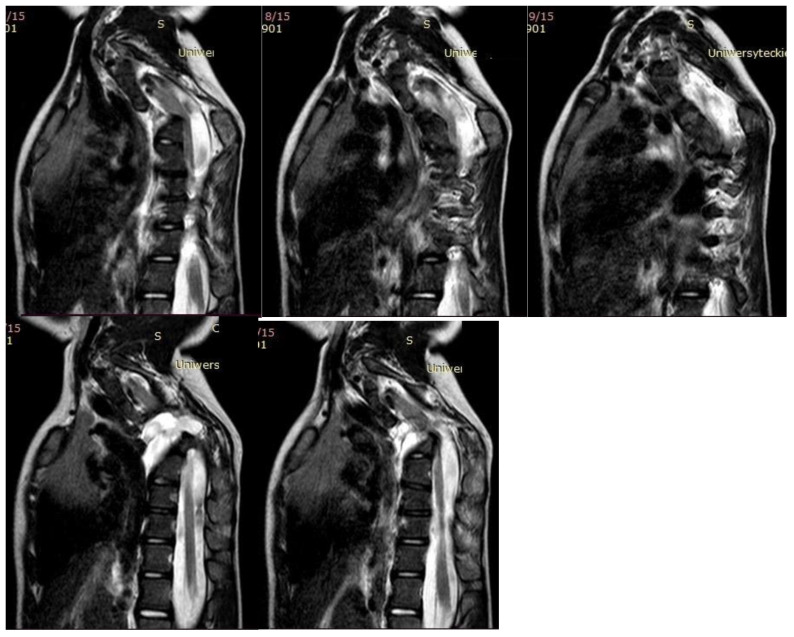
MRI of the five-year-old girl before surgical treatment.

Neurological examination before the surgical treatment indicated neurological function was intact. MRI and CT performed before the surgery showed multilevel mixed congenital spinal anomalies in the cervical and thoracic spine, without spinal cord pathologies (hemivertebrae, blocked vertebrae, Figure 2 and Figure 3). 

**Figure 4:**
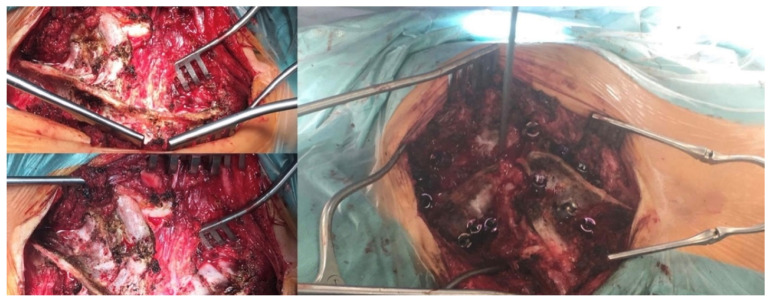
Posterior approach to the cervical and thoracic spine.

**Figure 5:**
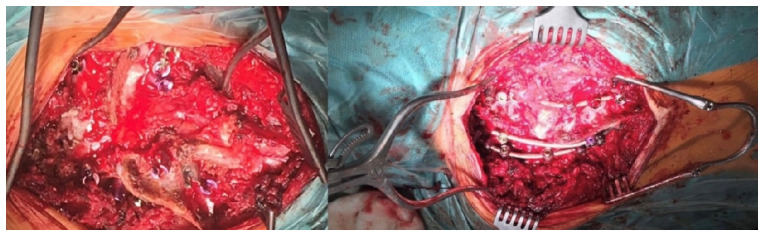
Posterior instrumentation and correction of C7–T9.

Angio-CT examination of blood vessels in the neck, chest, and abdomen showed no pathological structure. The patient underwent posterior vertebral column resection of T3–T4 (the blocked vertebrae) using the costotransversectomy approach (Figure 4 and Figure 5) with posterior pedicle screw stabilization and fusion between C7 and T9.

**Figure 6:**
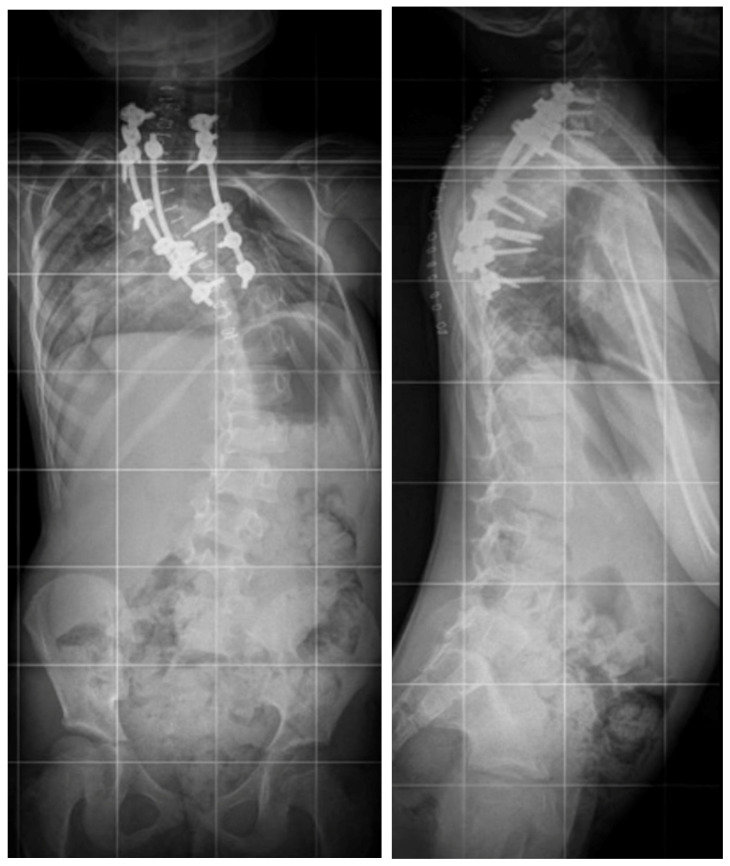
Standing X-rays of the five-year-old girl after surgical treatment via the posterior approach and VCR T3–T4.

Correction of the deformity included right-sided compression and left-sided distraction. The pleura remained intact during the surgery, and no chest tube was needed (Figure 6).

**Figure 7:**
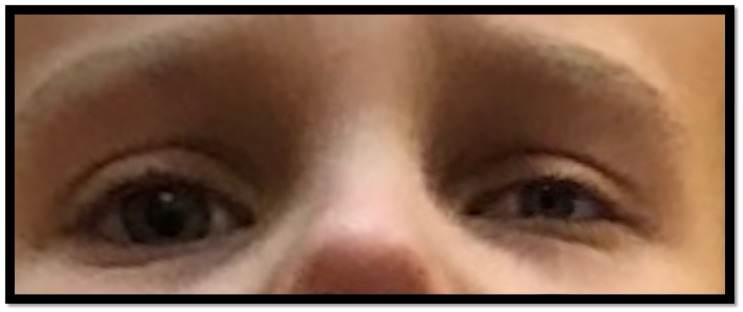
The child presented with worsening left-sided ptosis, miosis, and anhidrosis.

After successful surgery, on the same day, the child presented with worsening left-sided ptosis, miosis, and anhidrosis. No other symptoms were noted in the physical examination and neurological status examination. No blunt trauma occurred to her neck during surgery. No other symptoms or neurological deficits were noted, nor any headaches. Other than the triad of signs presented, all examinations were normal. The child was diagnosed with Horner’s syndrome, and we then sought to intensively diagnose the cause of the disease (Figure 7). 

**Figure 8:**
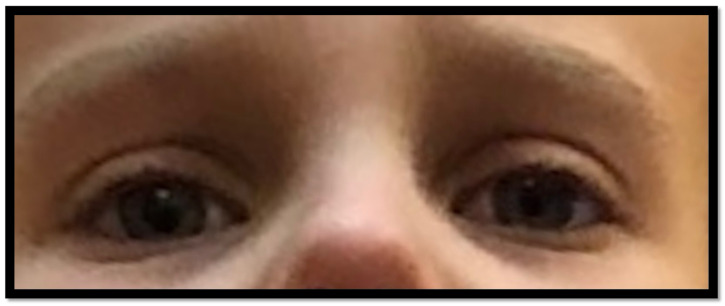
The child presented with full improvement after experiencing Horner’s syndrome.

We carefully reviewed the family’s medical history and found no cancer, neurological disease, stroke, or vascular disease. The imaging tests performed before the operation showed no changes in the lungs or the nervous system. We also noted no vascular anomalies. Given the lack of any clear pathologies, we began to consider intraoperative trauma. We suspected a hematoma in the brain could cause similar symptoms or a dissection of the carotid artery. We performed a Doppler ultrasound of the carotid arteries, computed tomography of the head and cervical spine, and magnetic resonance imaging of the head and cervical spine. These studies did not show pathologies that could explain the cause of the disease. The only possible cause of the symptoms of Horner’s syndrome was spinal correction of the deformity. The operation consisted of resection of the fused T3–T4 vertebrae, and then compression on the right side and distraction on the left side of the spine. Although we did not observe any changes in the monitoring of the spinal cord intraoperatively, the symptoms that occurred on the left side fit the clinical status of the patient. The child presented with HS due to distraction of the sympathetic chain at the high thoracic area and thus to the left eye. Since we did not find any other confirmation of Horner’s syndrome, the patient was discharged home on an elective basis, as after every elective spinal surgery. After six months of follow-up, the HS had fully resolved. At the four-year follow-up, the child was fully intact (Figure 8). No special treatments or rehabilitation were undertaken.

## 3. Discussion

The treatment of pediatric spinal deformities depends on their etiology. The gold standard in the treatment of idiopathic scoliosis is rehabilitation and corset treatment, and in the case of curvature progression despite the treatment above of over 50 degrees, Cobb-surgical treatment [15,16]. In the case of congenital scoliosis with vertebral segmentation disorders, conservative treatment and a brace do not tend to have a significant impact on the development of curvature, and surgical treatment is recommended [17]. HS classically consists of the symptom triad of miosis, ptosis, and anhidrosis. These clinical findings result from an interruption of sympathetic innervation of the eye, which can occur at different levels. The occurrence of this rare complication is associated with injury or interruption of the oculo-sympathetic nerve pathway, somewhere between its origin in the hypothalamus and destination in the eye. The fibers of the pathway originate from the posterolateral hypothalamus and descend uncrossed to the lateral midbrain, pons, medulla, and spinal cord in the cervical region to reach the C8–T2 (sometimes T4) spinal cord center [1]. Here, the connection with the preganglionic fibers takes place. Analysis of the entire fiber path depends on the location of the disturbance or damage. Thus, the causes of the appearance of HS can be central, preganglionic, or postganglionic. The localization of lesions causing HS is of great importance. First-order neuronal lesions are caused by central nervous system disorders, such as tumors, vascular occlusions, and disorders of the upper cervical spinal cord. Second-order neuronal lesions are caused by trauma to the brachial plexus, chest surgeries, metastases, and lung tumors. These injuries are most frequently involved in iatrogenic lesions (84%), compared with the three levels of the sympathetic pathway [2,3]. Third-order neuronal lesions are mostly caused by degenerative changes. Other causes include surgeries on the carotid artery or nearby structures, internal carotid artery dissection, and tumor extension [4]. 

### 3.1. Epidemiological Data

The etiology of HS in children and adolescents is not yet fully understood. Often, it is congenital or acquired [5]. In the available literature, a study conducted on a group of 73 pediatric patients showed that approximately 42% of HS cases were congenital in nature and occurred after undergoing surgical treatment of the chest, neck, or central nervous system. Additionally, the authors reported 15% of cases of acquired HS that were associated with brachial plexus injury, intrathoracic aneurysm, brain stem vascular malformations, spinal cord tumors, neuroblastoma, rhabdomyosarcoma, and embryonal cell carcinoma [6]. In the same study, the most common malignancy presenting with HS was neuroblastoma, and isolated HS was the first manifestation of neuroblastoma in 2% of cases. In a 1984 survey by Musarella et al., ophthalmic involvement occurred in 80 of 405 patients with neuroblastoma [7]. Bhate et al. [8] also highlighted the association between neuroblastoma and HS and the value of imaging studies. 

### 3.2. Spine Surgery

Perioperative etiological factors must also be considered. The study by Nasser et al. [12] showed that a high insertion point for a chest tube may cause compression of the stellate ganglion or post-ganglionic fibers, leading to ischemia and potential nerve injury due to neuropraxia of the second neuronal pathway. No complications were observed during left internal jugular central venous catheter insertion during surgery. Although it is extremely rare (0.4–2.5%) [14], a few studies have described HS cases secondary to epidural anesthesia following instrumented surgeries. We refer to case reports by Cowie et al. [1], who described HS secondary to epidural anesthesia following posterior instrumented scoliosis correction, and Yang J and Cho et al. [18], who described HS after spinal fusion secondary to epidural anesthesia. Transient HS has also been reported in several studies in patients who received epidural anesthesia, mostly after obstetric surgeries [19,20]. The explanation for epidurals and spinal anesthesia as a potential cause of HS may be that to perform this anesthesia, a local anesthetic must be injected near the spinal cord and nerve roots. Somatic sensory, motor, and autonomic nerve fibers are located in the thoracic and lumbar regions. Sympathetic outflow occurs in the range from T1 to L2. Sensory and autonomic fibers are smaller in diameter and can be blocked much more easily than large, fast-conducting motor fibers. In contrast, sympathetic blocks usually extend 1–2 levels higher than sensory blocks. Large variability in the location of the C8–T2 (sometimes T4) cilio-spinal center, coupled with unintended high blockage secondary to overdosing of local anesthetic or extensive spread, is the most likely and explainable cause of HS.

On the other hand, Mueller et al. reported HS after scoliosis correction, which was not caused by epidural anesthesia but was due to a mechanical cause, the placement of the implant and a hook located around the T4 vertebral epiphysis [9]. There are also studies demonstrating HS occurrence after anterior and posterior cervical spinal fusion and posterior hemivertebra excision [9,10,18]. Nevertheless, we were unable to find any reports of persistent HS as a complication of posterior hemivertebra T3–T4 resection during scoliosis correction surgery.

### 3.3. Diagnosis and Prevention

It should also be noted that there are some studies where pediatric HS cases were found to be idiopathic. George et al. noted no etiology in 70% of infants with HS diagnosed in the first year of life [21], and Smith et al. could not find an etiology in 35% of HS cases in children at mean five years of follow-up [22]. HS is very rare, has a subtle clinical picture, and is therefore often underdiagnosed [11]. All authors emphasized the role of early observation in patients with no or partial resolution of symptoms. It is necessary to deal with the potential long-term repercussions and effects. In the diagnostic process of a child with HS, computed tomography, magnetic resonance imaging, and pharmacological tests with cocaine should be used to confirm the clinical diagnosis. The pupil in Horner’s syndrome, compared to a healthy pupil, dilates less. Routine use of 1% hydroxyamphetamine in children with HS to distinguish preganglionic from postganglionic lesions is not recommended, as it is not always reliable, especially in younger patients. This contrasts with its usefulness in adults [6].

Prevention of acquired HS may be possible through familiarization with the procedures that may cause this complication. HS persistence may be observed on cranial, cervical, and vascular imaging. Most studies analyzing HS propose a systematic approach to lesion localization by means of accompanying signs and symptoms, followed by anatomical imaging with focused magnetic resonance imaging or computed tomography with angiography [23]. Some authors, like Reede et al., advocate determining whether HS is associated with first-, second-, or third-order neuronal damage and performing focused imaging using computed tomography or magnetic resonance imaging [24]. Computed tomography or magnetic resonance imaging of the brain, neck, chest, and thoracic spine are also needed to exclude additional causes of HS [4,25]. Therefore, Mahoney et al. [26] recommended that pediatric patients suspected of having HS should undergo a general physical examination and palpation of the neck, armpits, and abdomen. If HS is confirmed clinically or pharmacologically by testing, an MRI of the head, neck, and chest, with or without contrast, should be performed in addition to randomized urinalysis for HVA and VMA. Nevertheless, the best and most widely recommended protocol for evaluating children with HS of unknown etiology remains unclear. The early diagnosis of HS is extremely important because often, the usually benign or idiopathic causes of HS can be very serious and even fatal. Therefore, it is important to understand how to recognize, diagnose, and assess HS properly and promptly and to undertake the most appropriate management [27]. Ophthalmological examination provides sufficient evidence for HS. 

In our case, HS might have been due to injury to the sympathetic chain when vertebral column resection was performed, the osteotomy was closed without any anterior wall support by cage or mesh and the left-sided osteotomy underwent gentle distraction. We presume that the cause of HS was the stretch effect of surgery near the level T3–T4 bone block of the spine, although the complete preganglionic lesion should have occurred at T1–T2. In addition, the anatomy of the cervico-thoracic section has a complicated course of vertebral arteries, which may increase the risk of vascular damage. Therefore, we suggest that the course of vessels be properly diagnosed by CT angiography before planned surgical treatment. It is necessary to confirm the course of the vertebral artery in congenital defects of the spine, as in careful preparation in the case of preservation of the transverse opening at the level of the hemivertebral vertebra in CT images. The absence of intraoperative complications and cardiorespiratory impairment, combined with the delayed onset of blockage symptoms, are likely to support these explanations. Hered et al. [18] showed that direct trauma could have been one possible cause of HS in this study. The authors had performed instrumented posterior spinal fusion, but the patient did not experience any other neurological injury. The patient may have had an anatomic variant resulting in the sympathetic pathway exiting more distally than that described in a normal patient, allowing for injury in the instrumented thoracic spine. Although we have not been able to clearly identify the etiology in the current study, we report this case to highlight HS as a possible complication after posterior vertebral column resection in scoliosis correction surgery. We strongly believe that further investigations and identification of additional cases will contribute to a better understanding of this clinical condition and its prevention. 

## 4. Conclusions

Pediatric HS after posterior instrumented scoliosis correction surgery with posterior vertebral column resection of the thoracic spine is rare. The diagnosis of HS immediately after surgery may not be easy, particularly when the patient is sedated. The possible causes of HS should always be considered. Avoidance of high chest tube insertion, professional and mindful anesthesia care during anesthesia administration, and minimization of surgical dissection in the oculosympathetic nerve pathway may help minimize the potential risks of iatrogenic HS in children. Special care should be taken during corrective procedures using the compression-distraction technique in the high thoracic spine, which may lead to a direct injury. Symptom resolution is variable and may be delayed.

## Data Availability

Not applicable.
